# Order and change in art: towards an active inference account of aesthetic experience

**DOI:** 10.1098/rstb.2022.0411

**Published:** 2024-01-29

**Authors:** Sander Van de Cruys, Jacopo Frascaroli, Karl Friston

**Affiliations:** ^1^ Antwerp Social Laboratory, University of Antwerp, 2000 Antwerp, Belgium; ^2^ Department of Psychology, University of Turin, 10124 Torino, Italy; ^3^ The Wellcome Centre for Human Neuroimaging, University College London, London WC1N 3BG, UK; ^4^ VERSES AI Research Lab, Los Angeles, 900016, CA, USA

**Keywords:** psycho-aesthetics, curiosity, aha experience, predictive processing, active inference, neuroaesthetics

## Abstract

How to account for the power that art holds over us? Why do artworks touch us deeply, consoling, transforming or invigorating us in the process? In this paper, we argue that an answer to this question might emerge from a fecund framework in cognitive science known as predictive processing (a.k.a. active inference). We unpack how this approach connects sense-making and aesthetic experiences through the idea of an ‘epistemic arc’, consisting of three parts (curiosity, epistemic action and aha experiences), which we cast as aspects of active inference. We then show how epistemic arcs are built and sustained by artworks to provide us with those satisfying experiences that we tend to call ‘aesthetic’. Next, we defuse two key objections to this approach; namely, that it places undue emphasis on the cognitive component of our aesthetic encounters—at the expense of affective aspects—and on closure and uncertainty minimization (order)—at the expense of openness and lingering uncertainty (change). We show that the approach offers crucial resources to account for the open-ended, free and playful behaviour inherent in aesthetic experiences. The upshot is a promising but deflationary approach, both philosophically informed and psychologically sound, that opens new empirical avenues for understanding our aesthetic encounters.

This article is part of the theme issue ‘Art, aesthetics and predictive processing: theoretical and empirical perspectives’.

## 1. Introduction

How to account for the powerful effects of art on us? For example, what do people mean when they insist that a piece of art (be it literature, visual art or music) has helped them through difficult times? Clearly, they do not mean this in the literal sense of ‘helping’: art does not help like antibiotics do, eliminating the worldly cause of distress. Rather, art relieves distress by modifying our mental states—our construction of the brute facts of the world—and with that it opens up new opportunities for action. It provides the means for articulating, understanding and, ultimately, accepting or transforming our situation and ourselves.

Art does this not by painting a rosy picture of reality, nor by simply yielding to our prior expectations about the kind of world we should encounter. Tension, uncertainty and the violation of our expectations are part and parcel of good art; as has been noted since the earliest philosophical writings in aesthetics. Indeed, the genuine moments of discovery—that art enables—seem to demand tensions and uncertainties (*change*) as much as they demand closure and certainty (*order*). Moreover, the order reached through art is often an impetus for further forays into change: it acts as a self-validation that gives us the freedom to venture into a capricious and precarious world again, exploring new environments and ways of being (*change*). This renewed autonomous agency broadens horizons and (re)focuses one's actions and values. This is perhaps why our encounters with art are often also considered transformative, ethical experiences: they shape (freedom of) action.

In this paper, we try to shed new light on these aspects of our experiences of art and our aesthetic experiences more broadly, using the framework of predictive processing (PP; a.k.a. active inference). At first glance, this framework, which holds that biological agents are governed by the sole imperative to minimize uncertainty, seems squarely at odds with the possibility of rich aesthetic experiences of the kind just described. Indeed, it seems opposed to all the artistic, playful and creative pursuits that enlighten our existence. However, beneath this seemingly superficial and conservative principle, the framework has resources to illuminate crucial features of our aesthetic encounters, ranging from the most mundane to the most sublime.

## 2. The contours of aesthetic experience

Before we delve into the details of our proposal, we should clarify what we take our explanatory target to be. The first thing to note is that we consider art as providing or aiming to provide a particularly intense and satisfying experience, an experience that—although elicited by many non-artistic encounters—finds, in art, a paradigmatic and programmatic trigger: i.e. an ‘aesthetic experience’. The problem is then to flesh out the characteristic features of such an experience. This will occupy us for the rest of the paper, but some things can be noted at the outset. Based on Kantian intuitions, Shaviro describes the core of an aesthetic experience by drawing the crucial contrast with desire: ‘Desire is how the self projects itself into, and remakes, the world; aesthetic feeling is how the world projects itself into, and remakes, the self’ [1, p. 27]. This highlights a different direction of fit: while desire is about making the world fit the mind, aesthetic experience is about making the mind fit the world. An aesthetic experience grabs you, instead of it being about something you grab (because you need it). It is about being moved or touched [2], instead of being the mover of the world. Interestingly, this implies a receptivity—a readiness to adapt—but also something that is still beyond your receptivity, beyond what you can currently comprehend.

The emphasis on the world-to-mind direction of fit (remaking the self) also suggests aesthetic experience is not a distinct, exceptional kind of experience. One could say that life is a constant negotiation between moments when we reshape the world to our wishes, and moments when we just have to give way and cope with what the world serves us. In this minimal sense, there is an aesthetic dimension to any experience, and it is stronger the more we—our self and mental models of the world—are moved instead of being the ‘mover’. It also implies that aesthetics is fundamentally about restructuring our mind; that is, *learning* [3,4], though not necessarily a learning of the ‘cerebral’ kind, as we will see. This position (which some will recognize as having in Dewey [5] an important predecessor) implies that, just as we should see art as providing intensified aesthetic experiences of the kind that non-art can also provide, so we should see aesthetic experiences as presenting—in an enhanced way—certain fundamental traits of experience as such.

The tension between receptivity and incomprehensibility underlines four properties of aesthetic experiences that need to be addressed by our account. The first is that an aesthetic experience is a process, rather than a moment or the instantaneous appraisal of a static thing (an ‘artwork’). It is an interaction between object and subject, the temporal dynamics of which will need to be spelt out [2]. This paper is an attempt to characterize these dynamics from the ground up. Again, in the tradition of Kant's third Critique [6] and Dewey's *Art as Experience* [5], *the aesthetic* appears to designate the very development of an experience, which is a precondition for any further deliberate judgement or action. This inclusive, elementary sense of the aesthetic means that, as will become apparent later, there is a continuity between everyday experiences and full-blown, paradigmatic aesthetic experiences, the difference between the two being one of degree rather than structure.

Second, the tension between receptivity and incomprehensibility—and the emphasis on aesthetic experiences as processes—also make room for the often-noted contradictory ingredients in full-blown aesthetic experiences: the positive and the negative emotions [7], the disturbance and the harmony, the order and the change, the inward sense of closure and the outward sense of openness. Still, as we shall argue, none of these elements on their own are sufficient for an aesthetic experience.

Third, because of the tension between receptivity and incomprehensibility, individual differences in what we find aesthetically appealing are ubiquitous. The beholder's share [8,9] determines what one is receptive to and what one finds (in)comprehensible, and this inevitably shapes our aesthetic encounters. Something might not be conducive to a particularly powerful aesthetic experience for me, but it might be for you. I might even dismiss an artwork on one day, in one particular state of mind, but find it endlessly engaging on the next. This does not mean we cannot try to characterize the kind of objects that tend to invite aesthetic experiences (i.e. taking a ‘stimulus-oriented’ approach; [2,10]), but just that this analysis will always be incomplete. In the account proposed below, aesthetic experience is universal by its nature, but we are making an unwarranted projection when we say that aesthetic appeal is a property of the object.

Fourth and finally, the tension between receptivity and incomprehensibility suggests there is something ephemeral and unrepeatable to our aesthetic encounters. An aesthetic experience cannot be relived in the same way, because it consists in a change in one's relation to the object that prompted it (elaborated below). This does not mean that the same work cannot give rise to new aesthetic experiences, but rather that, as we will see, these will be new instances caused by different generative processes sharing a similar structure. It further means that we should distinguish between labelling something as aesthetically appealing [2] and going through an actual aesthetic experience. When we label or judge something as ‘aesthetically appealing’, ‘beautiful’ or ‘preferable’, it is not necessarily because—at this very moment—we enjoyed the characteristic generative process of aesthetic experience. It may simply be that we recognized that this stimulus (or a similar one) afforded that crucial process in the past. So even without going through an in-the-moment aesthetic experience, we can categorize things as beautiful or aesthetically appealing because we have learned the kind of processes they tend to evoke in us.

Having listed some of the desiderata of a theory of aesthetic experience, we can now move to introducing the neurocognitive framework that will act as our basis for such a theory, namely PP.

## 3. The epistemic arc

In a nutshell, PP holds that biological agents continually and largely implicitly generate top-down predictions to capture patterns (regularities) in their sensory inputs, and use the ensuing mismatches between those predictions and sensory samples (the bottom-up *prediction errors*^1^) to update and adapt the generative models that source our next predictions (for thorough but accessible introductions to PP, see [11–13]). In this way, PP provides the computational underpinnings for the radical constructivist maxim that we meet reality only in our failures (prediction errors), not in any absolute sense [14,15]. By minimizing those prediction errors, we infer the hidden causes that may have generated proximal sensations. For example, we infer the existence of objects (like ‘clouds’) in the world as the hidden cause, from the effects those objects tend to have on our senses. Importantly, our generative models are hierarchically structured, meaning that the predictions in lower-level regions serve as targets for predictions from higher levels, enabling those higher levels to tap into (and predict) more abstract regularities across space or time. For example, predictions about how a sentence will end inform—and are informed by—predictions about the next event in a narrative.

It follows from this picture that any experience, aesthetic or not, starts with a minimum of proactive engagement, in the form of predictions based on the context established by things previously inferred. Sensory data that matches our predictions recedes to the background, but sensory data that violate our predictions (increasing our uncertainty around their hidden causes) can grab our attention. They can become the (salient) cues for curiosity. Things we are curious about do not reveal everything immediately—they are shrouded in some uncertainty or violate our expectations in a given context [16]—but they offer an epistemic affordance that we are compelled to indulge [17].

But mere uncertainty, disarray or unpredictability is not enough to make us curious (think of the noise screen on TV). Rather, we need to feel—that is, have the implicit expectation—that we can make some progress in resolving our uncertainties. Recent computational theories of curiosity describe it as expected uncertainty resolution, equivalently known as expected information gain or learning progress [18–20]. These theories suggest that what sustains attention and drives us to explore a stimulus further is an epistemic promise: the promise that we will be able to resolve uncertainty, that if we work on our percept (i.e. actively test our perceptual hypotheses [21,22]) and let it work on us a little more, ‘things are going to make sense’ [23]. At minimum, the job of the artist is to sustain such promise, encouraging us to carry our exploration further.

Note that both the uncertainty we initially experience and the expectation that we can resolve this uncertainty are through-and-through subject-dependent:^2^ the specific mental (i.e. generative or world) model one applies to a situation determines what uncertainties are foregrounded and experienced. And whether one can expect to be able to cope with particular types of uncertainties similarly depends on whether one's model already captures some of the relevant regularities in this domain and so can help to make sense of it (including known unknowns). A piece of free jazz can be impossibly unpredictable for a novice (who may zone out or cut short the experience), but predictably unpredictable for the jazz lover.

Some accounts of curiosity have described our attraction towards those portions of our environment that assure uncertainty resolution as a sensitivity to the rate with which we will be able to reduce prediction errors (i.e. uncertainty) [24,25]. Such meta-expectations are not about predicting features of our environment but about predicting our own capacity to predict such features. If mental functioning is about minimizing prediction errors, predictions about our rate of prediction error minimization—within the current context or activity—measure how well we are coping and whether we need to invest more resources or not [26]. How do we acquire these meta-expectations? Probably in the same way we acquire all our expectations: through experience with similar contexts or activities—like playing an instrument, enjoying art or encountering stimuli of a particular type—and by experiencing how well we are able to reduce uncertainties there.

Of course, curiosity is expressed as *active* exploration: it is an urge to resolve uncertainty through *one's own actions* [17]. Consistent with this, in active inference, curiosity or expected information gain is an attribute of planned or counterfactual actions [13], quantifying their potential to forage sensory data that are diagnostic (salient) of the predictions one brings to bear to explain (the hidden causes of) a particular situation or stimulus. This so-called ‘epistemic foraging’ can range from eye movements to Internet searches.^3^ While it often involves an in-the-moment increase of prediction errors at lower rungs of the system, it is aimed at disclosing the structure of the world to reduce future prediction errors across hierarchical levels of abstraction. A common analogue here is a person who got lost and wants to return home but might go to a salient landmark first which, though it is *further away* from home (increasing prediction error), enables a return home with *more reliability* [18].

All this being probabilistic and the world being changeable, however, our information-seeking may actually increase prediction errors and uncertainty rather than reducing them. But if it is tuned well, uncertainty will generally be resolved, and prediction errors reduced (on average) under our continued sampling. Uncertainty that appears to be reducible piques curiosity, which, with time or epistemic actions, may lead to actual uncertainty reduction. When this happens, it usually feels good (See e.g. experimental studies by Ruan *et al*. [28] about the pleasure of uncertainty resolution).

Still, one has to keep in mind that for active inference, any mental process (including action) is assumed to be about reducing uncertainty, so even a mundane walk in the park is enabled by predictive models that minimize their uncertainty—relative to expected action programmes and associated predicted sensory consequences (for an overview on action as prediction, see [29]). To hone in on what kind of uncertainty resolution generates positive affect, we can again appeal to the idea of meta-expectations about the rate of uncertainty resolution. If the appetitive feeling of curiosity is linked to an *expected* rate of prediction error minimization, the *actual* rate may determine experienced affect after the epistemic act. Specifically, when uncertainty is reduced at a rate faster than expected, this might be marked by an intensely positive feeling [25,30–34]. There is a large psychological literature on this epistemic feeling, known as *Aha Erlebnis* or ‘insight’. In neurobiology, the recognition of uncertainty resolution has been linked to the dopaminergic discharges (of the sort associated with reward processing) [18] and has been unpacked in terms of ‘affective charge’ in the setting of affective inference [33].

**Figure 1.**
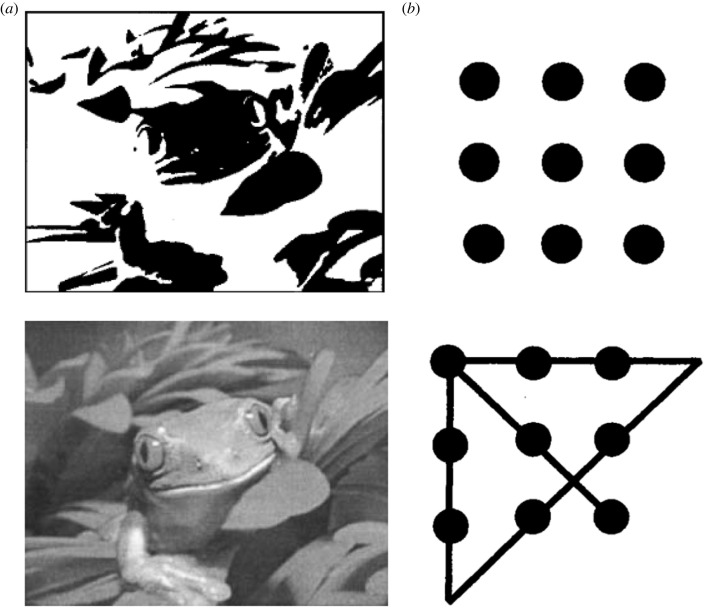
Two puzzles commonly used to induce aha experiences. (*a*) A Mooney or two-tone image, with the solution or source image (bottom). (*b*) The nine-dots problem [35] with solution (bottom). Participants are asked to connect the nine dots with four straight lines without lifting the pen from the paper.

Such aha experiences are usually preceded by an impasse, a phase of uncertainty in which we struggle to make sense of a situation or solve a problem despite some expended effort. The ‘puzzles’ that commonly induce an aha moment are usually simple enough on their face (e.g. a Mooney image with some recognizable shapes, three common words in the remote associates task, or nine dots in the nine-dots task; see examples in figure 1 [35–37]), so we are curious enough to engage with them and commit to epistemic acts to gather more information. We feel the solution is just around the corner.

If, with prolonged effort, we do not solve the problem, our meta-expectation about the rate with which we will be able to reduce prediction errors will be revised. When we finally and suddenly do settle on a good problem structuring (leading to the solution), we will have resolved uncertainty faster than expected, resulting in a pleasurable feeling of insight. Dubey *et al*. [38] indeed found evidence that the strength of the aha experience is causally linked to solving a puzzle faster than expected (i.e. a metacognitive prediction error).

Aha experiences may range from subtle perceptual insights (as in Mooney images) to insights in deliberate, cognitive problem-solving. They are notoriously hard to reliably elicit in the laboratory because they crucially depend on subjective factors, specifically a mental shift: a restructuring of the problem or stimulus or, equivalently, discovery of an underlying rule, symmetry or regularity. In terms of active inference, it is about selecting the hypothesis that best explains sensory input. The winning hypothesis or model will be as low in complexity and as accurate as possible, meaning that it will explain sensory evidence both well and parsimoniously.^4^ Settling on such a hypothesis implies an unexpected jump in precision or confidence in the newly found structure, as we indeed see in the confidence ratings associated with aha experiences [39]. Once a new structure is found, the problem often becomes trivial and automatized: the implicit actions that minimized uncertainty then have now become predominant ‘habits’. The fact that you were able to (re)construct the stimulus or solution yourself makes it feel more veridical and (ironically) out there in the world, independent of you: your grip on the world tightens and the world seems that more real and ‘at hand’ (see also, [40]). Indeed, one cannot ‘unsee’ the solution of the Mooney image. In our phenomenology, the solution becomes the stimulus, as it were (our struggle becomes transparent in Metzinger's sense [41]).

The aha experience also seems stronger when participants can discover the solution autonomously; that is, using their own epistemic agency instead of being informed by someone else. Epistemic agency is about the iterative testing of hypotheses or predictions based on different perceptual features in the input. More precisely, the process is one of relinquishing (down-weighting) one's preconceived, predominant (prior) hypothesis about the sensory data, and activating a less probable hypothesis that may show more explanatory promise, much like a scientist elaborating a novel hypothesis that underwrites her next experiment. Our tendency to assume and attach structure, rules or meanings to even partial perceptual cues and to readily situate those meanings in the external world instead of our mind regularly obstructs us from finding better, less complex and more evidenced solutions: we can sometimes be quite unscientific in our sensory exchanges with the world [42].

**Figure 2.**
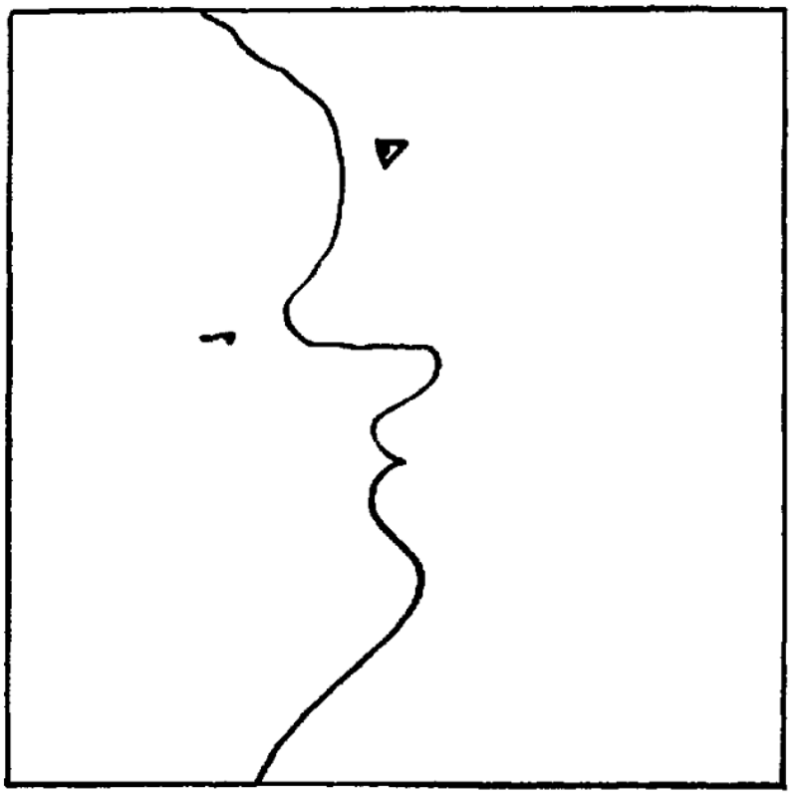
Ambiguous figure from Hebb [43]. Why do figures like this appeal? Absences can become meaningful (as obstacles or prediction errors) in a PP account. The simple line might make you expect little meaning in it. But that changes once you look a fraction longer, probably because your visual system registers that the line deviates (prediction error) from what you expect an average randomly drawn line looks like. This prediction error suggests an intention of the drawer, which in turn creates an expected reducibility of the prediction errors, which is fulfilled (after a brief search) with the discovery of a face. But this leaves some remaining errors: the one face has an odd contour, which leads to the discovery of a second face. Then, when one focuses on this new face, the other loses its ‘edge’ (a new error) because our visual system can only allocate it to one object, and so on. In a very simple stimulus, there are already micro-cycles of curiosity, epistemic action and discovery. Caption adapted from Van de Cruys *et al.* [25].

With the unexpected, pleasurable uncertainty resolution of the aha experience, we are able to close the *epistemic arc* that starts with curiosity and is followed by epistemic action(s) (See figure 2 for an example). The resulting emotion can range in positive intensity from ‘Oh, ok’ to ‘Aha!’. We hypothesize that the specific dynamics of uncertainty resolution and our expectations thereof will determine the intensity of the emotion. The experience of flow [44] might be due to a good match between expected and actual uncertainty reduction rates, where higher than expected rates may create more pronounced positive experiences as in the aha experience. The fact that our own actions underwrite the positive aha experience may indicate that affective valence specifically tracks the confidence (a.k.a. precision) with which actions can be expected to quickly minimize uncertainty [13,33].

## 4. Sense-making in art

Returning to art and aesthetic experience, it is now easy to see that the way artists capture people's interest and appreciation is through generating the opportunity for these epistemic arcs (i.e. these cycles of curiosity–epistemic act–aha experiences). Of course, the epistemic arc is an idealized experience. Curiosity might not be sated, epistemic acts may labour in vain (leading us to terminate the experience) and, depending on the dynamics of how we solve it (faster than expected or not), an aha experience may or may not round it off. But as a whole, the arc can be seen as a minimal unit of sense-making and a key component of the pleasure generated by art. To the extent that the experience afforded by an artwork to a particular observer approximates this ideal, one might argue, the artwork offers to that observer an aesthetic experience. Here, we might have a principled way to clarify Dewey's intuition that aesthetic experience presents in an enhanced way certain traits of experience as such.

**Figure 3.**
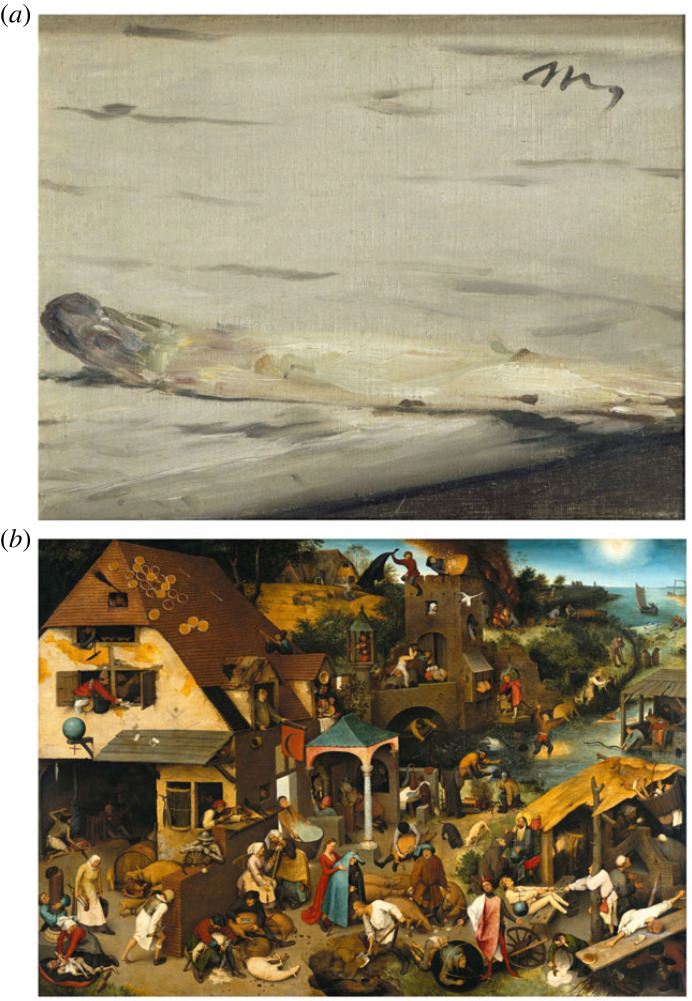
(*a*) *L'Asperge* by Édouard Manet. (*b*) *Nederlandse Spreekwoorden* by Pieter Bruegel de Oude. Both paintings allow a moment of puzzle-solving resulting in definite closure, either on the perceptual level (Manet: perceptual organization and discovery of the depicted object) or on the conceptual level (Bruegel: grouping of objects and discovering depicted proverbs). In both cases, top-down predictions (e.g. familiarity with the Dutch/Flemish language and cuisine) are of crucial importance in making discoveries, and thus enjoyment of the paintings. Manet increases uncertainty by violating grouping by similarity (lack of contrast between object and background), Bruegel by crowding the place and by the sheer weirdness (expectation violations) of the literal depictions of familiar proverbs. (Online version in colour.)

To provide us with such experiences, artists make use of prediction errors, and give them salience,^5^ thereby bestowing them with a promise of information gain or uncertainty reduction. This starts an epistemic arc. Because artworks are stimuli generated by humans with specific intentions and skills [48], we know there is a method to the ‘madness’, a hidden cause that we can infer to make the ‘deviant’ sensory array predictable. The hidden cause can be as simple as some objects depicted (as when we infer the rest of our inanimate surroundings), for example, when a few brushstrokes evoke the silhouette of a boat when placed in the right context (e.g. in a Monet painting). But the hidden cause can also be in *the way things are depicted*, the feelings and intentions that occupied the artist's mind when creating these specific physical inscriptions (as when we infer the thoughts and emotions of our social partners from their expressions). In art, the process of inference is often not as fluent as we are used to in navigating our everyday world (figure 3). Uncertainty resolution or sense-making is delayed by artists, and intentionally so; perhaps to extend the reach of the epistemic arc. This means that epistemic actions are necessary, even if on a short span, to arrive at a good generative model of the artwork (a model of how the stimulus was generated). The friction of prediction error creates the potential to (unexpectedly) make progress in structuring the stimulus, and, when this structuring happens by means of one's own epistemic (cognitive-behavioural) faculties, it sparks positive affect and situates the discovered structure in the outside world.

Now that we have sketched out the PP approach to aesthetic experience, let us try to defuse two objections that can be raised against it, namely, first, an excessive focus on the cognitive, problem-solving aspects, and second, an excessive focus on cognitive closure.

### (a) A cerebral slant?

A first charge that might be leveraged against the presented account is that it is too cognitivist: it focuses on mere ‘problem-solving’ on the largely implicit hypotheses our cognitive system continually formulates about the sensory barrage. And indeed, we described our engagement with the artwork as an ‘epistemic’ or information-seeking process, which seems to be a far cry from the deeply affective and existential experiences that art can engender (for other discussions on aesthetic cognitivism, see [49,50]).

However, for PP existential and epistemic concerns are intertwined from the start. To paraphrase the poet Paul Valéry: if something exists, it is producing future. To produce future, an organism has to embody a model of its environment—and, crucially, of the consequences of its own actions. PP formalizes the epistemic dynamics that allow the organism to do the existential work of resisting thermodynamic dispersion. If it is understood that uncertainty reduction is always only defined relative to the model embodied by the agent (i.e. by the model that the agent *is*), it becomes clear that this epistemic drive always has intrinsic existential relevance. By minimizing uncertainty an organism is self-evidencing: maximizing evidence for its own existence [51]. As the aphorism attributed to Novalis goes: ‘We look for the structure in the world—we are that structure.’ Exactly the same (provable) conclusion undergirded the early cybernetics movement [52].

As humans, the structures (or patterns) we do tune to and create to support life are varied and expansive. The patterns captured in our models range from simple perceptual regularities in our external environment, to regularities that characterize our internal milieu: the peculiar workings of our own bodily systems, and how the external environment shapes these interoceptive patterns [53,54]. The patterns can also be abstract stories we tell ourselves about what kind of person we are, unpacked into the behaviours we expect ourselves to engage in, and the kind of (social) environment we expect ourselves to be in. As Ramstead *et al*. [55, p. 233] point out: ‘generative models are normative models of “what ought to be the case, given the kind of creature that I am”’. Hence, the agent's models are not merely epistemic (representational) devices, but also normative and aspirational ones [56], even if constantly negotiated with the world.

Crucially, the epistemic arc and the associated ebbs and flows of uncertainty also take place relative to the hierarchical models that concern the dynamics of our interoceptive states and, on a higher hierarchical level, the self and its feelings we use to explain particular combinations of interoceptive and exteroceptive states [57–60]. Feelings emerge as high-level ‘empirical priors’^6^ or learned predictions that efficiently summarize regular packages of multimodal sensory flow, including the sensory flow caused by our own behaviour [57]. This is in line with the view that our feelings are inferred or constructed, rather than given [58,59]. Those ‘feeling’ predictions can then be readily applied to (recognized in) new instances in our own but also in others' behaviour (cf. theory of mind). Crucially, they can also be used to infer the hidden causes of inanimate products (expressions) of other humans’ behaviours like art. The pleasure is derived here from increasing attunement between the generative model of the artwork (as ‘proxy’ of the artist) and that of the perceiver.

This view of art aligns with widespread intuitions about the role of resonance in art which have been articulated effectively by Aristotle [61] and Tolstoy [62]. Art captures implicit regularities in our (affective) life that are rarely articulated in conscious, verbalizable concepts, even though they are an important part of our experience. Tolstoy writes: ‘To the recipient of a truly artistic impression it seems that he knew the thing before but had been unable to express it’ [62, p. 144]. Note the oblique reference to discovery (aha) and the unexpectedness of it. Internal (affective) patterns, experienced as utterly personal and idiosyncratic, *unexpectedly* find their evidence or validation, externally, in the world [63]. In this way, our active, epistemic engagement with art provides a unique, (quasi)social form of self-evidencing [51]: it provides evidence for (i.e. reduces uncertainty about) the existence of the self (model). Perhaps paradoxically, we might experience this as a temporary dissolution of the epistemic and existential boundary between ourselves and the outside world (or the other agent). As Tolstoy astutely observes [62, p. 197]: ‘A real work of art destroys, in the consciousness of the receiver, the separation between himself and the artist, nor that alone […] In this freeing of our personality from its separation and isolation, in this uniting of it with others, lies the chief characteristic and the great attractive force of art.’ On this view, the pleasure derived from encounters with art is due to this process of unexpected, *increasing* attunement or ‘fusion of horizons’ [64].

### (b) A fetish for closure?

Other critics of the presented account of aesthetic experience might contend that it puts too much emphasis on ‘completion’, ‘insight’ or ‘mastery’ and the associated momentary pleasurable outcome. However, with the way we have embedded insights or uncertainty resolution within an epistemic arc, we have already dispelled the notion that such completion can be attained in isolation from the dynamics of increasing and decreasing uncertainty. It is not the case that a ‘completed’ positive aesthetic experience always requires a temporally extended run-up (people can make snap aesthetic evaluations; [65,66]), but the ingredients of the epistemic arc should be present. This also implies that subsequent experiences with the same work will be faint copies of the original one (however, much we would like to revisit it) unless the work allows for new and different arcs.

Still, one could ask whether a failing model—mere uncertainty, mere dissonance, discrepancy or ambiguity—might be sufficient for an aesthetic experience, even without any resolution [67,68]. This question stands out especially for contemporary art that commonly challenges perceivers with dissonances and subversions (see e.g. Kesner [69], for a discussion of Dominik Lang's *EastWest* consisting of just ‘two holes in a cardboard wall’). Such works are regularly met with outright dismissal from their (potential) audience, due to frustration or boredom. Frustration may be felt because the epistemic arc (the effort of additional information-seeking) to get to the hidden cause is much longer than one expected or is accustomed to. It is about not finding the solution of the problem posed by the work (or indeed the problem that the work poses) *and* a lack of trust that there is something to figure out, more precisely: a low meta-expectation that there are reducible prediction errors in this context. Boredom is likely about the very belief that there is nothing more to the work, or that anything there *is* to it requires an investment of effort that is much greater than that which the artist made.^7^ Again, this can be seen as a breach of trust (the balance of effort in the communication that art is), considering that trust can be interpreted in a PP way as an expectation on the reliable reducibility of prediction errors: because a human made it, as a human I expect to be able to make sense of what they made. The pattern strictly does not have to be put in there by the artist, although the trust that *something* is put in there will keep us going (incidentally, this is a crucial difference with AI-generated art).

So as long as curiosity is sustained, ‘mere uncertainty’ can make for an aesthetic experience. Which means that this particular person, with their specific past experience with similar works or the same work just a moment ago, expects prediction errors to be reducible still. Another way to put this is that artists need to meet some of their audience some of the time, even if cultural learning may be necessary to bring the (predictive models of) perceivers to the ‘regime of reducibility’.

Still, when an artwork resists our ‘regularizing’ efforts, it may be that, in a ‘meta’ sense, the artist's statement (the generative idea behind the work) is about this very pattern (for example, our trust in the artist leading us to resolvable patterns). Artists are indeed very inventive in finding patterns in our failures in pattern-finding. Their object is not just the content of what is depicted but also the assumptions of our perceptual and cognitive, even art-historical sense-making systems [71,72]. As we pointed out above, art can concern patterns in the real or imagined world or your own (bodily) self's workings. More generally, the discovered pattern will usually be on a different level from that of the apparent ‘error’.

**Figure 4.**
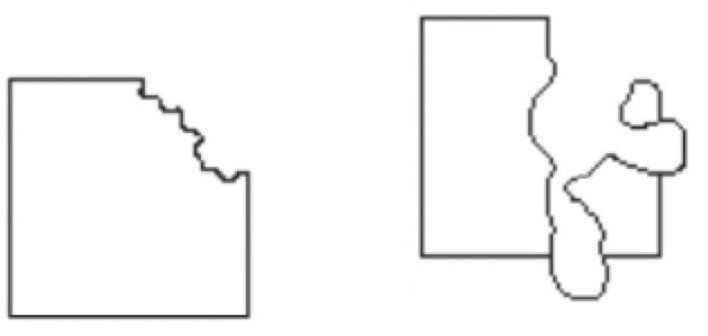
We immediately see not only shapes but also what happened to them (their causal history). Adapted from Pinna [73].

To take a very simple example, a square with a deformed corner will still be seen as a square (figure 4), but one to which something happened (e.g. a square with its corner melted or bitten off depending on the deformation; see [73] for more examples). Such *happenings* are patterns as well, in that we readily inferred and perceive (a plausible account of) the causal history of sensory inputs (a generative model) [74,75]. Art observers uncover similar happenings for new ‘errors’ in art, with more effort and so more risk (to leave the experience empty-handed) but also more pleasure when making unexpected (risky) predictive progress.

**Figure 5.**
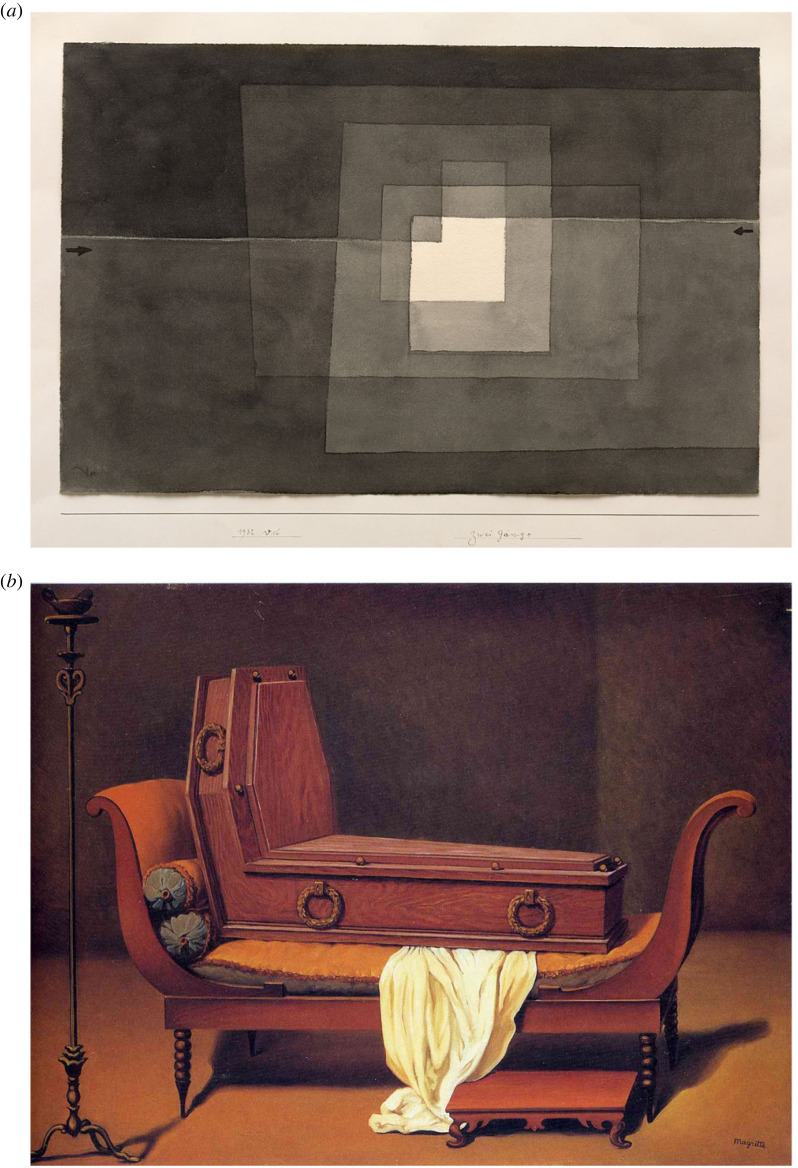
(*a*) *Zwei Gänge* by Paul Klee. (*b*) *Perspective: Madame Récamier de David* by Magritte. These works by Klee and Magritte illustrate that temporary discoveries are enabled by persisting incongruities that find no final resolution. On the perceptual level, in his paradoxical, multi-stable images, Klee explicitly and knowingly exploits the limitations of the eye, and its need for epistemic action to resolve uncertainty, as is apparent from this quote: ‘The limitation of the eye is its inability to see even a small surface equally sharp at all points. The eye must “graze” over the surface, grasping sharply portion after portion, to convey them to the brain which collects and stores the impressions’ [77, p. 33]. On the conceptual level, Magritte also invites inferences and epistemic actions by the very strangeness of a coffin on a lounger (again prior experience with the original by David or the other *Perspectives* by Magritte informs the search): is it a simple memento mori? Is it the symbolic death of a certain pictorial style or way of painting? Is it a humorous deconstruction of the ephemeral values of the upper class that commissioned these sorts of portraits? No interpretation is likely to settle the matter entirely (and luckily so, from our perspective too). (Online version in colour.)

In this hierarchical picture, closure on one level might bring openness on the next (as when identifying objects in a painting leads to questions about their relationship). Even if there is closure, this does not imply there is just one, definitive closure to the work (put there by the artist). Good art is often open-ended, which we now can understand specifically as allowing multiple arcs, multiple cycles of active (epistemic) inference. While instant closure destroys the aesthetic experience, the process towards closure, with the feeling of directionality that comes with it, is crucial and would be missed by defending a ‘mere uncertainty’ stance. Artists will intuitively provide a good ‘supply of structural indeterminacy’ in their works [76] because stabilization would mean the end of the inferential journey, and, with it, the end of pleasure and engagement with the object (figure 5). Openness and lingering uncertainty are, therefore, part and parcel of that difficult dialectic that the artist tries to maintain to poise us for predictive progress.

We suspect that the source of the ‘mere uncertainty’ objection may partly lie in the language used in the PP literature. When we speak of ‘explaining away prediction errors', the idea is not to remove or ‘tidy up’ those ‘unruly elements’, but rather to give them meaning as part of the general symbolic economy of the artwork. Indeed, prediction errors or dissonance in art provide the very source for new patterns to emerge [48]. Prediction errors just are the ‘newsworthy’ information from the sensorium that we need to account for; they are that which begs explanation and drives further learning (belief updating). With prediction errors, artists defamiliarize the world [78]: they give us a sense of how it was when we experienced the world for the first time, when we still had the greatest learning gains to make and the most patterns to discover.

A similar terminological misconception may arise for the concept of self-evidencing. This term emphatically does not imply a one-sided effort to make the world (i.e. artwork) conform to a static self. Indeed, we saw earlier that the ‘insight’ phase of the arc entails a form of restructuring of one's models in an effort to better align with the world. Self-evidencing just means to maximize the (marginal) likelihood of the sensations we sample from the world. It is to get a grip on the world. The twist offered by art (and possibly all encultured niche construction) is that architects and artists, musicians and poets afford us additional and very refined possibilities for self-restructuring and structuring of our sensed world.

Take for example the classic nine-dots ‘insight’ puzzle (figure 1) in which participants are asked to connect the nine dots with four straight lines without lifting the pen from the paper [35]. In such problems, one's background assumptions must be first recognized and dropped before the solution can appear [70]. After the struggle or impasse, dropping assumptions (i.e. reducing our model complexity) while gaining the power to explain existing or future data (finding solutions) provides faster-than-expected uncertainty resolution (aha experience).

In puzzles, these tensions and reliefs are merely about incidental patterns in the world, but in art they can involve assumptions or patterns that define us. Those higher-level priors organize large swaths of our behaviour and the sensory patterns we have come to expect, so there will be resistance (e.g. in the form of dismissal or avoidance of the artwork), unless new priors can be found through the artwork, which capture patterns in our environment or ourselves in a better or more comprehensive way. New self-related priors, primed by the conflicts in art, can validate (formerly conflicting) thought or action patterns, and past evidence generated by our own actions much better and so set in motion the deep self-transformation often considered as the highest forms of aesthetic encounters [79].

To conclude this part, it may seem that the epistemic arc approach to art explains too much. It does not allow us to draw very hard boundaries between genuine artworks and other cultural products like detective stories, jokes, Internet memes, puzzles, games [80], etc. In a deflationary way, all of these rely on epistemic arcs with obstacles, active information-seeking and relief, brought on by the manipulation of patterns and uncertainty. Arguably, there is indeed an aesthetic element in all these activities, but it is often an aesthetics of convergent closure: a closure that is indeed engineered in advance, a singular pattern for all to discover. There might be multiple arcs in a detective story, but they are typically all resolved in one particular way. In such plots, there may be diversions and uncertainty (gradual reveal of information), but the pieces are pre-processed, not self-paced and delivered to lead to one resolution,^8^ with minimal epistemic actions. By contrast, great art tends to stretch the arc in time and radiate out to multiple patterns.

What is the relation of this aesthetics of openness with the aesthetics of closure? Our reasoning here, and in previous accounts of aesthetic experience, tended to look backward [81] on the arc from the position of closure: relative to some ‘expected set’—given one's predictions or prior preferences—it feels good to reduce errors, especially if unexpectedly. This component is crucial, but, as we will see now, it is limiting and narrowing. Instead of looking back, can we also look ahead beyond the arc?

## 5. Looking ahead to extend the arc

The problem with an explanation of aesthetic phenomena merely built on aha experiences or ‘closure’ is that such cognitive engagements are self-effacing. In PP, all cognition is expectation-driven, which necessarily narrows perception. We perceive relative to those set points we happen to put on the world. A resource-limited, goal-driven system has to narrow its perception in this way, but there is a real danger of getting entrapped in our own constructions: closing our epistemic arcs too soon or too permanently, such as in false dissociations (there is a rich literature on this phenomenon in computational psychiatry; e.g. [82–84]). While aha experiences are caused by restructurings of our patterns of thought and action, they also strongly anchor those new patterns, as can be seen from high confidence ratings associated with these experiences, even if they turn out to be false ones [39]. Epistemic actions, so central in PP, may seem to save the day here, because they lead to direct encounters with prediction errors (see above), rather than their avoidance. But even if, being curious, one expects to gain information through some actions, this is still anchored on the models or hypotheses one can formulate about the causes of sensory inputs. An aha experience may even have a self-sealing effect, in the sense that the closure reached implies that any further thoughts and explorations (epistemic acts) are terminated [85–87], at least with respect to the specific matter that started the epistemic arc.

However, there seems to be a wider, more *beneficial* effect on subsequent information-seeking as well. A recent empirical study reports that experiencing an aha increases one's tolerance for uncertainty, as measured with a risky decision-making task [88]. We hypothesize that the aha experience, being a mark of past epistemic success, gives rise to a generalized expectation that uncertainty will be swiftly reducible (positive expected rate of uncertainty reduction; see also [3]). Because the completed arc involved one's (epistemic) actions, coming actions receive a confidence boost that seems to reach beyond the particular setting in which the aha emerged. This might allow one to ‘stay with uncertainty’ instead of seeking greedy closure, so the next arc might be longer. This may be how art grows arcs, gradually raising the cognitive investment—and associated gains—in a surmountable way. Longer arcs require but also build trust. A similar dynamic of building trust through tension-relief cycles can be seen in play behaviour [89].

Compare this with Internet memes as the paradigmatic case of short epistemic arcs.^9^ Memes contain easily surmountable disfluencies—quick tension-relief arcs—but no less feeling of truth or completion. The aha in memes also points to restructuring or learning, but it is a form of trustless learning, because the challenge is minimal and the restructuring often surfaces beliefs (patterns) one already possessed, but were not activated initially by the meme ‘set-up’. So there is little new understanding. As meme-like conversation becomes the norm, driven by its success on trustless, attention-starved social media, we may (meta-) learn to expect such quick return on cognitive investments, and disengage from anything that has a longer arc. So when we credit art with providing the basis for humanistic aspirations such as deferred judgement and nuanced, mindful reasoning, we probably recognize its capacity to train us to avoid premature epistemic closure, to be at ease in uncertainty and to continue our epistemic foraging.

## 6. Freedom forward

Let's return to the somewhat simplistic example of the nine-dots puzzle. To open the hypothesis space that contains the solution, it might be said that the beauty of the solution has to be sacrificed: the strong implicit prior that the lines drawn will stay within the perfectly regular, symmetric dots-frame. However, only by violating that prior, another aesthetic is enabled: the renewed freedom of action that leads to the solution. Although stripped from all existential connotations, this illustrates another principle we see in aesthetic experience. At least since Kant and Schiller, aesthetic experiences have been associated with freedom, in many different but related ways. The creator of a beautiful object, says Kant, does not merely follow established rules but freely establishes them [6]; the beautiful object, in turn, is said to display a certain freedom from external laws (‘beauty is freedom in appearance’, says Schiller [90, p. 152]), which in turn awakens a similar freedom in the perceiver, whose cognitive faculties are put in a state of ‘free play’ [6] or who is led to assume a kind of volitional openness, characteristic of playful behaviour [91]. Schiller even goes as far as claiming that ‘It is only through beauty that man makes his way to freedom’ [91, p. 90]. These Kantian and Schillerian ideas still inform present-day discussions in aesthetics, often in connection with other key notions such as those of autonomy and disinterestedness [48,92,93]. Arguably, then, freedom should play a role of some importance in a good account of our aesthetic experiences.

Where does this freedom come in within the active inference framework? Active inference is very explicit about how to choose actions (policies), and plan, when looking ahead, going beyond the reduction of prediction errors encountered in the here and now (this is sometimes called planning as inference [13,94,95]). Put briefly, it holds that we should choose those actions that minimize expected free energy or uncertainty.^10^ Without going into formal detail, expected free energy comprises an epistemic component—is the envisioned action my best way to resolve uncertainty about how my observations are caused?—and a pragmatic one—is the action my best way to fulfil my prior preferences? [13,18,99]. Scoring our potential action sequences for expected uncertainty naturally implies a balance between exploring (epistemic value or expected information gain) and exploiting (pragmatic value or expected utility: fulfilling one's goals).

Put briefly, as long as there is uncertainty about the relations between actions, inferred causes and observations, those actions with epistemic value greater than the pragmatic value of alternative actions will win out [18]. Interestingly, if one removes the ambiguity of those mappings, and one removes the prior preferences as well, ‘the only remaining imperative is to maximize the entropy of observations (or states)’ [13]. In this case, expected uncertainty is best minimized by sampling across all options (also called uncertainty sampling): to act so as to increase the dispersion (entropy) of attainable states. In other words, we expect to ‘keep our options open’. Under those specific circumstances of low uncertainty about the structure of your world, and no pressing prior preferences to attend to, ‘surprise can be minimized when an agent selects a policy [action sequence] that increases the likelihood of visiting new states' [100]. Empirically, Rens *et al*. [101] recently showed that human choice behaviour was indeed better accounted for by a model in which humans were not only maximizing expected utility but also increasing the availability of options. This availability of options also caused greater feelings of freedom in people. In another study, Navarro *et al*. [102] show that people are averse to the loss of options in a dynamic multi-armed bandit task.

It might seem paradoxical to say that an agent that is fundamentally geared towards the minimization of uncertainty or surprise also strives to *maximize* entropy (uncertainty) via its actions. The subtle but important distinction to grasp here is that the maximization concerns the relative entropy of the agent's beliefs, before and after acting: namely, maximizing information gain and reducing uncertainty about states of affairs [13]. Conceptually, minimizing uncertainty translates to explaining and predicting observations as accurately as possible, while avoiding commitments to overly specific explanations or assumptions [13,14,103], for which there is little evidence. In machine learning and statistics, a failure to comply with the implicit maximum entropy principle [104] results in ‘overfitting’. In short, while past observations need to be rendered as predictable as possible, in order to predict future observations with minimal error (minimize uncertainty in the long run) it is important not to commit to beliefs that are overfitted to past observations, but cast the widest net possible allowed by past evidence.

In action selection, this translates into sampling observations in a way that creates the *largest change* in beliefs. Observations are only meaningful if they shift our beliefs, or change in our mind in a meaningful way (i.e. afford information gain). If not, the input is uninformative and can be ignored [14]. Hence, the drive to maximize the number of hidden causes experienced through our actions is a key component of self-evidencing. An alternative perspective on the interplay between information and goal seeking is to decompose the imperatives for action into *ambiguity* and *risk*. Ambiguity minimization seeks out unambiguous mappings between inferred causes and observations, while minimizing risk minimizes the difference between anticipated outcomes (our prior preferences or goals). Note how active inference captures the essential tension—and the compromise to be found—between, on the one hand, excessive stability, namely overly constrained or canalized behaviour that threatens resilience (the capacity to deal with future changes) and, on the other hand, excessive dispersion or plasticity that threatens the physical integrity of the organism [13,105,106]. Or, in Whitehead's words: ‘The art of progress is to preserve order amid change, and to preserve change amid order. Life refuses to be embalmed alive’ [107, p. 339].

With this basic sketch of how to ‘look ahead’ with active inference, we can try to put some computational flesh on the somewhat enigmatic but intuitive ideas connecting aesthetic experience to freedom formulated by Kant and Schiller among others. In active inference, the value of freedom is an integral part of uncertainty minimization and specifically comes into play when two conditions are met. First, prior preferences recede into the background, a (riskless) condition that might capture what is often referred to in the philosophical literature as the *disinterestedness* characterizing our aesthetic experiences [108]: the encounter with art usually takes place when more primary needs are satisfied or at least are not at the forefront. Second, the ambiguity or uncertainty about how inferred causes (including our own actions) conspire to form our observations has to be reduced as well. As we saw above, aha experiences mark such moments of sudden clarity about the structure of the world: moments in which uncertainty is resolved. These two conditions release important constraints of the optimization function (expected free energy) for future action selection, and presumably free us up to go for a more radical, free exploration. As discussed before, even an aha experience as such seems to allow us to embrace more uncertainty than usual.

In practice, this may be expressed as a choice for unlikely (lower probability), less prepotent hypotheses or policies (action sequences) for the situation at hand, and be experienced as renewed freedom of action. There is disinterestedness when there is no pressing need to organize your sensorium in determinate fashion, and this leaves you free of exploring different possible organizations. While admittedly speculative, this reasoning opens a way to give substance to some of the more ineffable and profound features of aesthetic experiences. Indeed, art (like psychotherapy [109]) is said to renew autonomous agency and the motivation to explore a precarious world (and novel ideas). As a good case in point, think of the overwhelming aesthetic experience when arriving on a mountaintop overseeing a marvellous landscape. The unique epistemic insight, after our intense epistemic act of climbing the mountain, is fused with the unexpected opening of action affordances, an enormous vista for planning our next moves.

But where natural beauty does this in concrete space, art can do this in mental space. Hence, the sense of expansion and empowerment [110], the sense of gaining options to interact with and control our environment, sometimes associated with the highest aesthetic experiences (see also Nietzsche's idea of art as the sublimated will to power [111]). It may just be the phenomenology of what it is like to be in those specific ‘regions’ of free energy evaluation. This is where actions and values are opened up, where we do not just *approach* our goals and values by optimizing relative to what we already want and expect, but rather *learn what can be valuable* in *the first place* [112]. This is indeed what autotelic creatures, agents that create their own goals, arguably do [113]. For this, we need to be in a state where we can afford perturbations in our environment and in our internal models [114], to safely expand our hypothesis spaces. Indeed, we cannot use our current hypotheses (expected values) or expected information gain to learn about the unknown unknowns [115].

Art, like play, is, therefore, about facilitating a ‘sandboxing’ mode of active inference that allows us to relax some of the usual constraints in the computation of expected uncertainty. However, once the radical novelty has been allowed to enter, the standard tools to test viability of new constructs (epistemic and pragmatic value) kick in again, and cause the part of the aesthetic pleasure described in the first half of our paper. Art may be a way of bracketing well-trodden inferential processes, to try on new hypotheses [116,117], even new forms of agency or *selves* [80], shielded from the constraints that usually stymie these before they get the chance to accumulate evidence [118]. Because that accumulation of evidence for a previously unlikely construct—the unexpected self-evidencing—feels good. Again, it is neither the horizon-expanding nor the narrowing, nor the order nor the change, which sustains art (and life) but a cyclical interaction of the two.

The conditions of this aesthetic and creative mode, with its receptivity to uncertainty, will need to be examined in more detail. Some answers might be found in the particular shape of the generative models that we are able to maintain, specifically their hierarchical structure and counterfactual temporal depth [119,120]. The hierarchical organization of these models will substantially reduce the search space for novel hypotheses and can guide random search to most informative regions [121]. Further, confident (high precision) higher-level predictions could accommodate more variability in lower-level empirically learned priors and so allow more uncertainty in observational data to filter through (see example above about ‘happening patterns’ to a square). Such more abstractly formulated models can also be applied to new domains (analogical thinking).

Models with counterfactual temporal depth allow us to start from arbitrary assumptions and ‘dry-run’ their implications, in an inferentially isolated way, shielded from the usual constraints (priors). Uncertainty can be allowed to exist longer here. In sum, all of these ‘architectural’ model properties enable a more canalized, contained way of introducing useful uncertainty in the system [25]. But there are more situation-, trait- and state-dependent ways as well for our cognitive system to self-organize instabilities [122], centring around phenomena like curiosity, aha, trust and emotion/mood, which we know affect our openness to uncertainty, and which, as we hope to have shown, can be cast in terms of active inference.

## 7. Conclusion

We have seen that aesthetic experiences stem from unexpected validations of our model of the world (i.e. of the model that we *are*), validations that increase the challenge (uncertainty) one can engage with. It is about the *change towards order* (the uncertainty reduction in the arc) and the *order towards change* (a secure basis to open new options in freedom). It is about the unexpected reassurance that one's emotional dynamics are to be expected (normal) given one's reference frame (prior beliefs, goals, etc.), thereby rendering one's experiences predictable and meaningful, rather than aberrant, irrational or unpredictable. But it is also about what this ‘relief’ enables in terms of further, longer encounters with the *not-me*, with the yet-to-be-modelled. The process of attunement with your environment feels good, but it requires this encounter, which, when it throws up surmountable challenges (reducible prediction errors), builds trust. Only in interaction can we settle on the right, negotiated challenges that build trust: the room for making new (prediction) errors. We do so in play, conversation, therapy [123], music-making or in the interaction-by-proxy in art. Here, active inference appropriately celebrates the classical confluence of the epistemic, the aesthetic and the ethic (freedom of action).

And yet, at this point, the active inference account of art may feel as merely a unified and more precise reformulation of what eminent scholars of aesthetics (mentioned along the way) have more evocatively described. However, aside from unification, the active inference framework brings a promising but deflationary approach to complex experiences. While most theories on aesthetic experience start from the multifaceted richness of those experiences and describe the different cognitive and emotional components that seem to contribute to those experiences (perception, attention, memory, classification, mastery, metacognition, etc.; [124,125]), a PP approach starts with the simplest, general-purpose elements. The angle here is to gauge how far we can get in *building up* towards that rich complexity, following Feynman's adage that we do not understand that which we cannot create. Indeed, one major advantage is that those simplest elements (e.g. hidden causes, prediction errors, etc.) can be captured precisely in mathematical equations, and can be tentatively mapped to neural circuitry and physiology [13].

For experimental aesthetics, another implication is that one does not necessarily need to study actual artworks as stimuli to better understand aesthetics. This eliminates our problem of the lack of experimental control on those stimuli, and on the internal models that participants bring to bear on them, and hence any uncertainty experienced or reduced. The active inference approach is a welcome reminder that shedding more light on component processes such as epistemic acts and aha experiences can be just as informative in understanding aesthetics (see also [126]). What is specific to art might be in the interplay of those components as laid out above, more than in any of the generic components as such. Finally, we hope that the presented account may help close the notorious gap [127] between what we mostly study in experimental aesthetics—mere preferences—and what we proclaim to study: rich and deeply engaging aesthetic experiences, soaked with mixes of strong emotions and sublime undertones, that may change our outlook in life.

## Data Availability

This article has no additional data.

## Endnotes

1 Prediction error can be read as surprise or, in information theory, self-information. This means that the average prediction error is the average self-information or entropy—a measure of uncertainty.

2 Although we write ‘uncertainty that we experience’, note that uncertainty does not need to be prominent in the actual experience of curiosity. We talk about (resolvable) uncertainty here largely in the subpersonal sense, dependent on the models we view the world through. We do not experience the subpersonal generative process leading to curiosity, but only its results, as the ‘direct’ experience of the epistemic affect that we call curiosity. So one could say curiosity is what it feels like when recognising resolvable uncertainty.

3 Interestingly, active inference does not make a strict difference between overt and covert (mental) actions. A search for information can be internal, in the sense of selecting particular prediction errors by turning up their gain or precision [27].

4 Technically, Bayesian belief updating, and model selection, try to maximise model evidence (a.k.a. the marginal likelihood of some data under a model). Crucially, log model evidence can always be expressed as accuracy minus complexity. This means we are compelled to find accurate explanations for data that are as simple as possible (c.f. Occam's principle or Jaynes' maximum entropy principle).

5 Salience in active inference is read as the expected information gain or epistemic affordance. This is sometimes referred to as intrinsic motivation or value, especially in machine learning and neurorobotics [20,45–47].

6 Empirical priors are a technical term for the beliefs or expectations at intermediate levels in hierarchical (i.e. deep) models. They play the role of likelihoods from the point of view of higher levels and priors on lower levels. Crucially, empirical priors are always updated in the face of new data.

7 Technically, in active inference, effort scores the degree of belief updating [70]. By definition, the epistemic arc bakes in the right amount of effort, in the sense that resolving uncertainty just is belief updating.

8 Assuming the author can keep your attention until the ‘season finale’.

9 As another example of the pleasure caused by quickly resolvable arcs, one could think of the mildly unpredictable ‘hooks’ in pop songs that immediately resolve into more predictable rhythms/melodies.

10 Technically, PP is rooted in the Bayesian brain [96] and the Free Energy Principle [13]. Variational free energy is an upper bound on the negative logarithm of Bayesian model evidence [-lnP(õ|m)], known as surprise/surprisal. Under some simplifying assumptions, free energy is just the average of prediction errors or uncertainty [13,97,98].
